# Selective δ-Opioid Receptor Agonist, KNT-127, Facilitates Contextual Fear Extinction *via* Infralimbic Cortex and Amygdala in Mice

**DOI:** 10.3389/fnbeh.2022.808232

**Published:** 2022-02-21

**Authors:** Ayako Kawaminami, Daisuke Yamada, Shoko Yanagisawa, Motoki Shirakata, Keita Iio, Hiroshi Nagase, Akiyoshi Saitoh

**Affiliations:** ^1^Laboratory of Pharmacology, Department of Pharmacy, Faculty of Pharmaceutical Sciences, Tokyo University of Science, Chiba, Japan; ^2^International Institute for Integrative Sleep Medicine (WPI-IIIS), University of Tsukuba, Ibaraki, Japan

**Keywords:** fear memory, extinction, δ-opioid receptor, amygdala, infralimbic cortex, PTSD

## Abstract

Facilitation of fear extinction is a desirable action for the drugs to treat fear-related diseases, such as posttraumatic stress disorder (PTSD). We previously reported that a selective agonist of the δ-opioid receptor (DOP), KNT-127, facilitates contextual fear extinction in mice. However, its site of action in the brain and the underlying molecular mechanism remains unknown. Here, we investigated brain regions and cellular signaling pathways that may mediate the action of KNT-127 on fear extinction. Twenty-four hours after the fear conditioning, mice were reexposed to the conditioning chamber for 6 min as extinction training (reexposure 1). KNT-127 was microinjected into either the basolateral nucleus of the amygdala (BLA), hippocampus (HPC), prelimbic (PL), or infralimbic (IL) subregions of the medial prefrontal cortex, 30 min before reexposure 1. Next day, mice were reexposed to the chamber for 6 min as memory testing (reexposure 2). KNT-127 that infused into the BLA and IL, but not HPC or PL, significantly reduced the freezing response in reexposure 2 compared with those of control. The effect of KNT-127 administered into the BLA and IL was antagonized by pretreatment with a selective DOP antagonist. Further, the effect of KNT-127 was abolished by local administration of MEK/ERK inhibitor into the BLA, and PI3K/Akt inhibitor into the IL, respectively. These results suggested that the effect of KNT-127 was mediated by MEK/ERK signaling in the BLA, PI3K/Akt signaling in the IL, and DOPs in both brain regions. Here, we propose that DOPs play a role in fear extinction *via* distinct signaling pathways in the BLA and IL.

## Introduction

Heightened fear memory and its abnormal processing, such as dysfunction in extinction learning of fear memory, have been implicated in the pathophysiology of posttraumatic stress disorder (PTSD) and anxiety disorders (Blechert et al., 2007; de Quervain et al., 2017). Extinction learning is an inhibitory learning process by which a fear response developed in a life-threatening event is reduced after long or repeated retrieval of fear memory, in the absence of the life-threatening event (Walker et al., 2002). Therefore, extensive efforts have been made to develop pharmacological agents that facilitate fear extinction, and to establish novel agents for PTSD and anxiety disorders medication. To date, several drugs have been reported to facilitate extinction, such as D-cycloserine, a partial agonist of NMDA receptors (Hofmann et al., 2019); ketamine, a glutamate NMDA receptor antagonist (Girgenti et al., 2017; Feder et al., 2020); and WIN55212-2, a cannabinoid CB1 receptor agonist. In particular, D-cycloserine facilitates fear extinction in animals (Walker et al., 2002) and humans (Mataix-Cols et al., 2017). However, this drug has a serious side effect of potentiating the fear response by reconsolidating conditioned fear memory in rodents (Lee et al., 2006; Yamada et al., 2009). Therefore, a drug without such side effects is required in clinical use.

Growing evidence indicates that the several brain regions that participate in the process of fear extinction overlap with the regions involved in the process of fear memory retrieval, such as the amygdala (AMY), hippocampus (HPC), and medial prefrontal cortex (mPFC). These brain regions are also known as the target sites of action of drugs that influence fear extinction (Tovote et al., 2015). For example, the basolateral nucleus of the AMY (BLA) is involved in the acquisition of extinction memories (Herry et al., 2006), and the HPC is thought to mediate the contextual gating of extinction (Hobin et al., 2006). In addition, mPFC is an interconnected brain structure and plays a role in extinction of conditioned fear (Senn et al., 2014). In particular, the prelimbic cortex (PL) and infralimbic cortex (IL) have been reported to have opposing roles in the regulation of fear memory, with PL being active during fear memory retrieval and IL being active to suppress the fear response (i.e., fear extinction) (Senn et al., 2014).

The δ-opioid receptor (DOP) is a G-protein-coupled receptor widely distributed in brain regions involved in emotion processing (Saitoh and Nagase, 2018). Consistent with its distribution, activation of DOP produces robust antidepressant-like and anxiolytic-like effects in rodents (Saitoh et al., 2004, 2011, 2013; Nagase and Saitoh, 2020). In this context, we previously reported that a selective agonist of the DOP, KNT-127, facilitates contextual fear extinction in mice (Yamada et al., 2019). However, the brain regions and molecular mechanism(s) that mediate the extinction-facilitating action of KNT-127 remain elusive. Therefore, in this study, we first investigated the site of action of KNT-127 using local administration of the drugs into the BLA, HPC, PL, and IL.

Several reports have suggested that phosphorylation of mitogen-activated protein kinase (MAPK)/extracellular signal-regulated kinase (ERK) in the BLA, HPC, and mPFC is involved in the enhancement of extinction (Herry et al., 2006; Fischer et al., 2007; Girgenti et al., 2017). For example, administration of the MEK/ERK inhibitor to the BLA (Herry et al., 2006) or HPC (Fischer et al., 2007) increased the level of freezing behavior during extinction sessions. Interestingly, we previously showed that subcutaneous administration of KNT-127 before extinction training increased the phosphorylated forms of ERK1/2 (p-ERK1/2) in the BLA and HPC, not in the mPFC (Yamada et al., 2019). In addition, it has also been suggested that phosphorylation of the mechanistic target of rapamycin (mTOR) and its upstream kinase, protein kinase B (Akt), in the mPFC is upregulated by ketamine and resulted in facilitation of fear extinction (Girgenti et al., 2017). Therefore, we examined whether these two signaling pathways in the fear circuits contribute to the effect of KNT-127 on fear memory regulation.

## Materials and Methods

### Animals

Male C57BL/6J mice (6–8 weeks old, purchased from Tokyo Laboratory Animals Science, Tokyo, Japan) were used for the behavioral experiments. The mice had free access to food and water in an animal room maintained at 23 ± 1°C with a 12-h light–dark cycle (the lights were switched on automatically at 8:00 a.m.). The mice were kept in this environment for at least 1 week before the experiments. The study was conducted in accordance with protocols approved by the Institutional Animal Care and Use Committee of the Tokyo University of Science (approval nos. Y19032, Y20020, and Y21002).

### Stereotaxic Surgery

Stereotaxic surgery was performed as previously described (Yamada et al., 2009). First, mice were anesthetized with a mixed solution of medetomidine (0.75 mg/kg), midazolam (4.0 mg/kg), and butorphanol (5.0 mg/kg) dissolved in saline. Then, the guide cannula (5.0 mm, AG-5 or 3.0 mm, AG-3; Eicom, Kyoto, Japan) was implanted over the BLA (coordinates AP, −1.46 mm; ML, ±3.2 mm; DV, 4.3 mm from the bregma), HPC (coordinates AP, −3.28 mm; ML, ±3.0 mm; DV, 4.5 mm from the bregma), PL (coordinates AP, +1.78 mm; ML, ±0.3 mm; DV, 2.25 mm from the bregma), and IL (coordinates AP, +1.70 mm; LM, ±1.70 mm; DV, 2.0 mm from the bregma angled at 30°) (Paxinos and Franklin, 2001; Xiong et al., 2017) and was fixed in place with dental cement. Finally, a dummy cannula (Eicom) was inserted in the guide cannula to avoid clogging. After the surgery, atipamezole (medetomidine antagonists) was administered to the mice at a dose of 0.75 mg/kg. Then, the mice were monitored for ≥5 days for recovery (normal eating, drinking, and defecation).

### Drug Infusions

KNT-127 and naltrindole (NTI) were synthesized by Dr. Nagase (University of Tsukuba, Ibaraki, Japan) and were dissolved in phosphate-buffered saline (PBS). U-0126 was purchased from FUJIFILM Wako Chemicals Corp. (Osaka, Japan) and was dissolved in 5% DMSO and 6% Tween 80 and diluted to a concentration of 1.0 μg/0.2 μl in 0.1 M PBS. First, KNT-127 or PBS was administered each 30 min before reexposure 1 (extinction training). Then, dummy cannula was removed, and microinjection cannula (Eicom) connected to infusion tube, syringe (Hamilton Company Inc., Reno, NV, United States), and the injection pump (KD Scientific Inc., KDS Legato 111, Muromachi Kikai Co., Ltd., Japan) was placed into the guide cannula. The tip of the microinjection cannula extended 0.5 mm beyond the guide cannula. Drugs were bilaterally infused at a rate of 0.2 μl/min for AMY (25 and 50 ng/mouse), HPC (50 ng/mouse), PL (50 ng/mouse), or IL (50 ng/mouse). For all brain regions, the total volume of infusion was 0.4 μl/mouse, and the total time of infusion was 1 min. After infusion, microinjection cannulae were left in place for 2 min to allow the drug to diffuse. Second, to examine the involvement of DOPs, PBS or NTI was administered 30 min before PBS or KNT-127 administration. The dose of NTI (250 ng/0.2 μl) was based on a previous study (Randall-Thompson et al., 2010; Sugiyama et al., 2018). Third, to examine the involvement of the MEK/ERK signaling in fear extinction, vehicle (VEH) or U-0126 was administered 30 min before saline or KNT-127 subcutaneous administration. The dose of U-0126 was based on a previous study (Miller and Marshall, 2005; Wells et al., 2013). Finally, to examine the involvement of the PI3K/Akt pathway in contextual fear extinction, vehicle (VEH) or LY294002 was administered 30 min before saline or KNT-127 subcutaneous administration. The dose of LY294002 was based on a previous study (Slouzkey and Maroun, 2016). At the end of the behavioral test, mice were anesthetized with isoflurane and microinjected with 0.2 μl per side of fast green. The brains were sliced using a linear slicer PRO7N (DOSAKA EM, Kyoto, Japan). Cannula location was confirmed and if cannula tip was outside of the target region, the animal was excluded from the analysis.

### Contextual Fear Conditioning Test

Contextual fear conditioning, extinction training, and memory testing were conducted in a conditioning chamber (20 cm × 20 cm × 33 cm, LabDesign, Ibaraki, Japan). The lateral and rear walls were made of opaque plastic. The chamber floor consisted of 19 stainless steel rods (4-mm diameter), spaced 1 cm apart and wired to a shock generator (ENV-414, Med Associates, St. Albans, VT, United States). The chamber was cleaned with 70% ethanol before and after each trial. On the conditioning day, the mice were trained with eight conditioning trials. Each trial consisted of a 1-s, 0.8-mA footshock, with an intertrial interval of 30 s. The mice were allowed to explore the chamber for 60 s before conditioning began, and they remained in the chamber for 30 s after the last conditioning trial. Twenty-four hours following the conditioning, the mice were microinjected with PBS or KNT-127. After 30 min, the mice were reexposed to the conditioning chamber for 6 min (extinction training, reexposure session 1). Twenty-four hours after reexposure session 1, each mouse was placed back in the conditioning chamber, and a 6-min test was performed (test, reexposure session 2). During each session, no footshock was given, and the mouse was observed every 1 min by a trained observer to assess its freezing behavior *via* a monitor connected to a video camera system mounted over the experimental chamber. Fear memories at the test session were assessed and expressed as a percentage of the time.

### Data Analysis

The data are expressed as the means ± SEM. One- or two-way analysis of variance (ANOVA) was used to compare more than two groups. *Post hoc* individual group comparisons were made using the Bonferroni’s test for multiple comparisons. The Student’s *t*-test was used for comparisons between two groups. Analyses were performed with GraphPad Prism 7 (GraphPad Software, San Diego, CA, United States); *p* < 0.05 was considered to indicate statistical significance.

## Results

### KNT-127 Reduced the Contextual Fear Memory *via* δ-Opioid Receptors in the Basolateral Nucleus of the Amygdala

**FIGURE 1:**
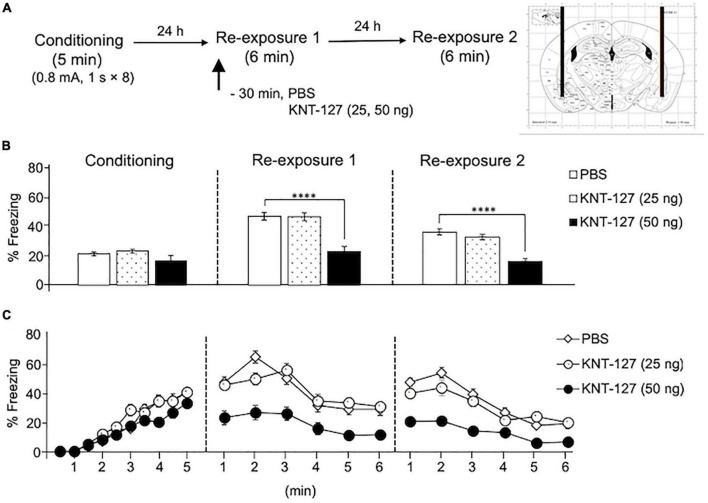
Intra-BLA administration of KNT-127 significantly reduced the contextual fear memory. **(A)** Experimental design. Mice were bilaterally administered with PBS (*n* = 16) or KNT-127 (*n* = 11 for 25 ng, *n* = 13 for 50 ng) into the BLA 30 min before reexposure 1 (6 min). Twenty-four hours after reexposure 1, a fear memory was tested in the same context for 6 min (reexposure 2). **(B)** Freezing rates of mice during conditioning, reexposure 1, and reexposure 2. KNT-127 significantly reduced freezing rates during reexposures 1 and 2. **(C)** Time course of the freezing rates of mice during conditioning, reexposures 1 and 2. Data were expressed as mean ± SEM. ****p < 0.0001 for comparisons between PBS group by one-way or two-way ANOVA and *post hoc* Bonferroni’s test.

To investigate whether KNT-127 acts in the BLA to facilitate extinction, we administered mice with various doses of KNT-127 (25 and 50 ng/mouse) 30 min before the reexposure to the conditioning chamber on day 2 (reexposure session 1) [PBS (*n* = 16) or KNT-127 (*n* = 11 for 25 ng, *n* = 13 for 50 ng); Figure 1A]. Figure 1B shows the total freezing rates of mice in each session. In reexposure 1, intra-BLA administration of KNT-127 at doses of 50 ng, but not at 25 ng, significantly reduced the freezing rate (one-way ANOVA: *F*_(2,37)_ = 22.44, *p* < 0.0001; *post hoc* Bonferroni’s test: *t* = 0.05237 for 25 ng; *t* = 6.079, *p* < 0.0001 for 50 ng) (Figure 1B). In reexposure 2, intra-BLA administration of KNT-127 significantly reduced the freezing rate at 50 ng, but not at 25 ng, when compared to PBS (one-way ANOVA: *F*_(2,37)_ = 28.12, *p* < 0.0001; Bonferroni’s test: *t* = 1.168, *p* = 0.5008 for 25 ng; *t* = 6.293, *p* < 0.0001 for 50 ng; Figure 1B). Figure 1C shows the time course of changes in freezing rates of mice in each session. Two-way repeated-measures ANOVA (drug × time) revealed significant main effects of the drug (*F*_(2,37)_ = 23.78, *p* < 0.0001), session (*F*_(2,74)_ = 71.22, *p* < 0.0001), and interaction between drug and session (*F*_(4,74)_ = 9.292, *p* < 0.0001) (Figure 1C). These results suggest that KNT-127 exerts fear-reducing effect by acting on the BLA.

**FIGURE 2:**
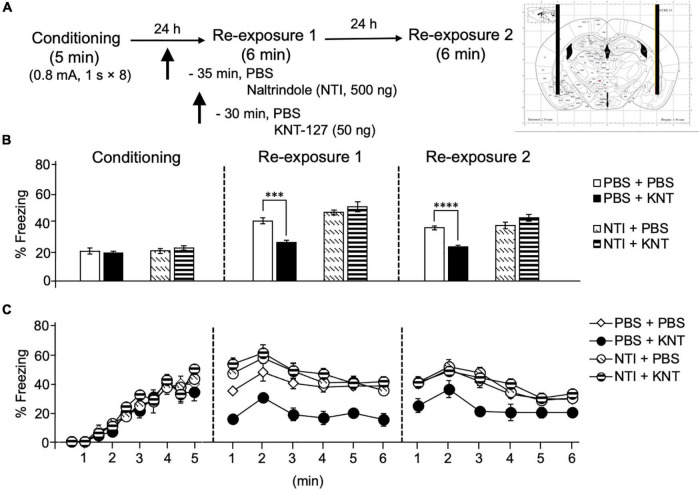
Intra-BLA administration of the DOP antagonist abolished the freezing reduction by KNT-127. **(A)** Experimental design. Mice were bilaterally administered with PBS or KNT-127 (50 ng) into the BLA 30 min before reexposure 1 (6 min). Five minutes before injecting these drugs, PBS or a DOP antagonist, naltrindole (NTI), was administered similarly (35 min before reexposure 1). Twenty-four hours after reexposure 1, a fear memory was tested in the same context for 6 min (reexposure 2). **(B)** Freezing rates of mice during conditioning, reexposures 1 and 2. I abolished the effects of KNT-127 during reexposures 1 and 2. The number of mice in each group was as follows: *n* = 9 for PBS and subsequent PBS (PBS + PBS), *n* = 6 for PBS and subsequent KNT-127 (PBS + KNT), *n* = 8 for NTI and subsequent PBS (NTI + PBS), and *n* = 8 for NTI and subsequent KNT-127 (NTI + KNT). **(C)** Time course of the freezing rates of mice during conditioning, reexposures 1 and 2. Data are expressed as the means ± SEM. Data were expressed as mean ± SEM. ***p < 0.001, ****p < 0.0001 for comparisons between KNT and NTI treatment groups by one-way or two-way ANOVA and *post hoc* Bonferroni’s test.

We also examined the effect of NTI, a DOP antagonist, on the contextual fear reduction by intra-BLA KNT-127. We administered PBS or NTI (500 ng/mouse) to mice in the AMY 30 min before administration of PBS or KNT-127 (50 ng/mouse) (*n* = 9 for PBS + PBS, *n* = 6 for PBS + KNT-127, *n* = 8 for NTI + PBS and *n* = 8 for NTI + KNT-127) (Figure 2A). Figure 2B shows the total freezing rates of mice in each session. In reexposure 1, NTI restored the freezing rate of mice that was reduced by KNT-127, and NTI itself did not affect the freezing rate (one-way ANOVA: *F*_(3,27)_ = 17.44, *p* < 0.0001; *post hoc* Bonferroni’s test: *t* = 4.236, *p* = 0.0014 for PBS + PBS vs. PBS + KNT-127; *t* = 1.826, *p* = 0.4740 for PBS + PBS vs. NTI + PBS; *t* = 1.146, *p* > 0.9999 for NTI + PBS vs. NTI + KNT-127; Figure 2B). In reexposure 2, NTI also abolished the effect of KNT-127 (one-way ANOVA: *F*_(3,27)_ = 17.78, *p* < 0.0001; *post hoc* Bonferroni’s test: *t* = 4.809, *p* = 0.0003 for PBS + PBS vs. PBS + KNT-127; *t* = 0.6357 for PBS + PBS vs. NTI + PBS; *t* = 2.043, *p* = 0.3053 for NTI + PBS vs. NTI + KNT-127; Figure 2B). Figure 2C shows the time courses of freezing rates for each session. Two-way repeated-measures ANOVA (drug × session) revealed significant main effects of session (*F*_(2,54)_ = 153.5, *p* < 0.0001), drug (*F*_(3,27)_ = 21.88, *p* < 0.0001) and interaction between drug and time (*F*_(6,54)_ = 7.25, *p* < 0.0001; Figure 2C). Therefore, these results suggest that the action of KNT-127 in the BLA is mediated through the DOPs.

### Intra-Basolateral Nucleus of the Amygdala Administration of MEK Inhibitor Antagonized the Fear-Reducing Effect of KNT-127

**FIGURE 3:**
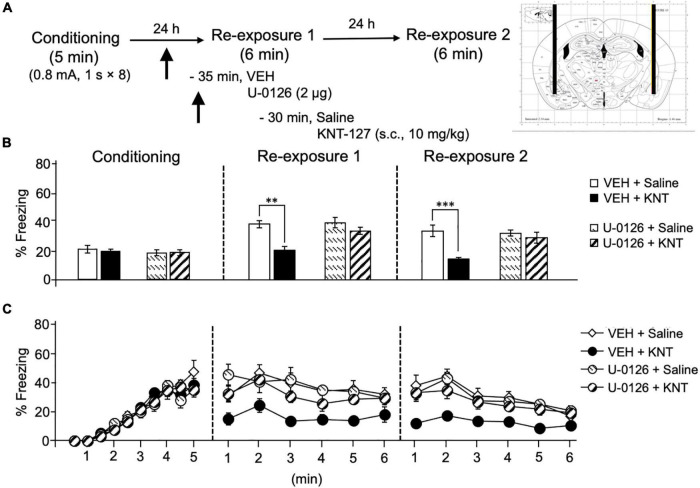
Intra-BLA administration of the MEK/ERK inhibitor blocked the fear-reducing effect of KNT-127. **(A)** Experimental design. Mice were subcutaneously administered with saline or KNT-127 (10 mg/kg) 30 min before reexposure 1 (6 min). Five minutes before injecting these drugs, a vehicle or a MEK inhibitor, U-0126, was administered bilaterally into the BLA (35 min before reexposure 1). Twenty-four hours after reexposure 1, a fear memory was tested in the same context for 6 min (reexposure 2). **(B)** Freezing rates of mice during conditioning, reexposures 1 and 2. U-0126 abolished the effects of KNT-127 during reexposures 1 and 2. The number of mice in each group was as follows: *n* = 6 for vehicle and subsequent saline (VEH + saline), *n* = 7 for vehicle and subsequent KNT-127 (VEH + KNT), *n* = 7 for U-0126 and subsequent saline (U-0126 + saline), and *n* = 7 for U-0126 and subsequent KNT-127 (U-0126 + KNT). **(C)** Time course of the freezing rates of mice during conditioning, reexposures 1 and 2. Data are expressed as the means ± SEM. Data were expressed as mean ± SEM. **p < 0.01, ***p < 0.001 for comparisons between KNT-127 and U-126 treatment groups, by two-way ANOVA and *post hoc* Bonferroni’s test.

Based on our previous result that phosphorylation level of ERK1/2 in the amygdala was upregulated by systemic KNT-127 and reexposure of mice to the conditioned context, we next examined the effect of U-0126, a MEK inhibitor, on KNT-127-induced contextual fear reduction. We administered VEH or U-0126 (2 μg/mouse) to mice in the BLA 30 min before s.c. administration of saline or KNT-127 (10 mg/kg, s.c., *n* = 6 for VEH + saline, *n* = 7 for VEH + KNT-127, *n* = 7 for U-0126 + saline, and *n* = 7 for U-0126 + KNT-127; Figure 3A). Figure 3B shows the total freezing rates of mice in each session. U-0126 restored the freezing rate of mice that was reduced by KNT-127, and U-0126 itself did not affect the freezing rate (one-way ANOVA: *F*_(3,23)_ = 8.467, *p* = 0.0006; *post hoc* Bonferroni’s test: *t* = 4.096, *p* = 0.0027 for VEH + saline vs. VEH + KNT-127; *t* = 0.2505 for VEH + saline vs. U-0126 + saline; *t* = 1.36, *p* > 0.9999 for U-0126 + saline vs. U-0126 + KNT-127; Figure 3B). In reexposure 2, U-0126 also abolished the effect of KNT-127 (one-way ANOVA: *F*_(3,23)_ = 9.648, *p* = 0.0003; *post hoc* Bonferroni’s test: *t* = 4.684, *p* = 0.0006 for VEH + saline vs. VEH + KNT-127; *t* = 0.4073 for VEH + saline vs. U-0126 + saline; *t* = 0.7419 for U-0126 + saline vs. U-0126 + KNT-127; Figure 3B).

Figure 3C shows the time courses of freezing rates for each session. Two-way repeated-measures ANOVA (drug × session) revealed significant main effects of each session (*F*_(2,46)_ = 40.35, *p* < 0.0001), drug (*F*_(3,23)_ = 9.88, *p* = 0.0002) and interaction between drug and time (*F*_(6,46)_ = 5.348, *p* = 0.0003; Figure 3C). These results suggest that the action of KNT-127 is mediated through the MEK/ERK pathway.

### KNT-127 Reduced the Contextual Fear Memory *via* δ-Opioid Receptors in the Infralimbic

**FIGURE 4:**
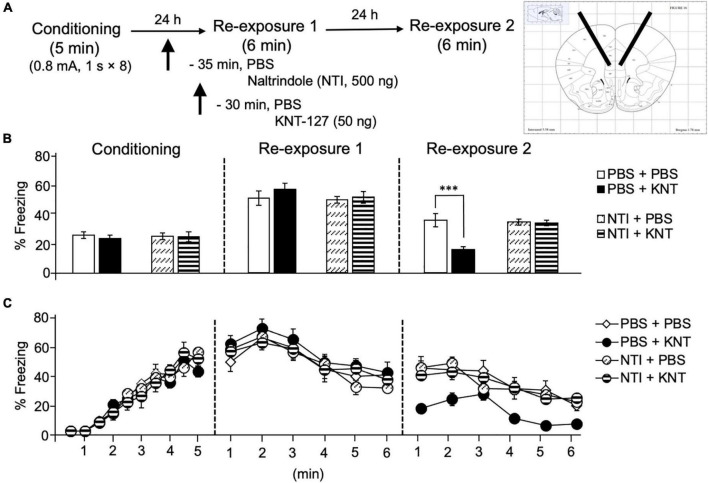
Intra-IL administration of KNT-127 significantly and DOP-dependently reduced the contextual fear memory only in reexposure 2. **(A)** Experimental design. Mice were bilaterally administered with PBS or KNT-127 (50 ng) into the IL 30 min before reexposure 1 (6 min). Five minutes before injecting these drugs, DOP antagonist naltrindole (NTI) was administered similarly (35 min before reexposure 1). Twenty-four hours after reexposure 1, a fear memory was tested in the same context for 6 min (reexposure 2). **(B)** Freezing rates of mice during conditioning, reexposures 1 and 2. NTI abolished the effects of KNT-127 during reexposures 2. The number of mice in each group was as follows: *n* = 6 for PBS and subsequent PBS (PBS + PBS), *n* = 7 for PBS and subsequent KNT-127 (PBS + KNT), *n* = 8 for NTI and subsequent PBS (NTI + PBS), and *n* = 9 for NTI and subsequent KNT-127 (NTI + KNT). **(C)** Time course of the freezing rates of mice during conditioning, reexposures 1 and 2. Data are expressed as the means ± SEM. Data were expressed as mean ± SEM. ***p < 0.001 for comparisons between KNT and NTI treatment groups by one-way or two-way ANOVA and *post hoc* Bonferroni’s test.

Next, we aimed to examine the contribution of another key brain region in the fear extinction circuit, IL, to the fear-reducing effect of KNT-127. We administered PBS or NTI (500 ng/mouse) to mice in the IL 30 min before administration of PBS or KNT-127 (50 ng/mouse, *n* = 6 for PBS + PBS, *n* = 7 for PBS + KNT-127, *n* = 8 for NTI + PBS and *n* = 9 for NTI + KNT-127; Figure 4A). Figure 4B shows the total freezing rates of mice in each session. NTI in the IL produced no significant effect in reexposure 1 (one-way ANOVA: *F*_(3,26)_ = 0.6968; Figure 4B), but NTI abolished the effect of KNT-127 in reexposure 2 (one-way ANOVA: *F*_(3,26)_ = 13.14, *p* < 0.0001; *post hoc* Bonferroni’s test: *t* = 5.166, *p* = 0.0001 for PBS + PBS vs. PBS + KNT-127; *t* = 0.3433 for PBS + PBS vs. NTI + PBS; *t* = 0.1928 for NTI + PBS vs. NTI + KNT-127; Figure 4B). Figure 4C shows the time courses of freezing rates for each session. Two-way repeated-measures ANOVA (drug × session) revealed significant main effects of session (*F*_(2,52)_ = 115.8, *p* < 0.0001) and interaction between drug and time (*F*_(6,52)_ = 5.671, *p* = 0.0001), but not drug (*F*_(3,26)_ = 1.22, *p* = 0.3223; Figure 4C). These results suggest that the action of KNT-127 in the IL is mediated through the DOPs. These results suggest that KNT-127 exerts fear-reducing effect by acting on the DOPs in the IL.

### Intra-Infralimbic Cortex Administration of PI3K Inhibitor, but Not MEK Inhibitor, Antagonized the Fear-Reducing Effect of KNT-127

**FIGURE 5:**
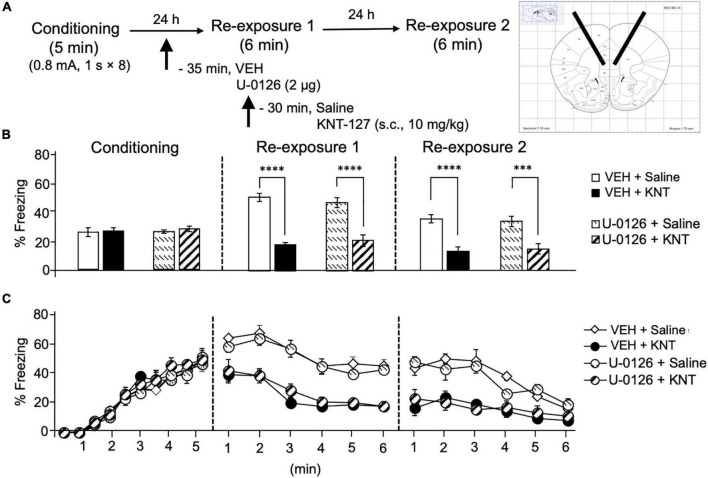
Intra-IL administration of MEK/ERK inhibitor did not affect the fear reduction induced by KNT-127. **(A)** Experimental design. Mice were subcutaneously administered with saline or KNT-127 (10 mg/kg) 30 min before reexposure 1 (6 min). Five minutes before injecting these drugs, a vehicle or a MEK inhibitor, U-0126, was administered bilaterally into the BLA (35 min before reexposure 1). Twenty-four hours after reexposure 1, a fear memory was tested in the same context for 6 min (reexposure 2). **(B)** Freezing rates of mice during conditioning, reexposures 1 and 2. U-0126 abolished the effects of KNT-127 during reexposures 1 and 2. The number of mice in each group was as follows: *n* = 5 for vehicle and subsequent saline (VEH + saline), *n* = 5 for vehicle and subsequent KNT-127 (VEH + KNT), *n* = 5 for U-0126 and subsequent saline (U-0126 + saline), and *n* = 5 for U-0126 and subsequent KNT-127 (U-0126 + KNT). **(C)** Time course of the freezing rates of mice during conditioning, reexposures 1 and 2. Data are expressed as the means ± SEM. Data were expressed as mean ± SEM. ***p < 0.001, ****p < 0.0001 for comparisons between KNT-127 and U-126 treatment groups, by two-way ANOVA and *post hoc* Bonferroni’s test.

Our previous finding shows that the phosphorylation of ERK1/2 in the mPFC was not induced by subcutaneous administration of KNT-127 and following reexposure session 1 (Yamada et al., 2019). Therefore, we confirmed whether MEK/ERK signaling in the IL contributes to the fear-reducing effect of KNT-127. To this end, we examined the effect of U-0126, a MEK inhibitor, on KNT-127-induced extinction-facilitating effect. We administered VEH or U-0126 (2 μg/mouse) to mice in the IL 30 min before administration of saline or KNT-127 (10 mg/kg, s.c., *n* = 5 for VEH + saline, *n* = 5 for VEH + KNT-127, *n* = 5 for U-0126 + PBS, and *n* = 5 for U-0126 + KNT-127; Figure 5A). Figure 5B shows the total freezing rates of mice in each session. U-0126 in the IL produced no significant effects of KNT-127 in reexposure 1 (one-way ANOVA: *F*_(3,16)_ = 31.24, *p* < 0.0001; *post hoc* Bonferroni’s test: *t* = 7.532, *p* < 0.0001 for VEH + saline vs. VEH + KNT-127; *t* = 0.8167 for VEH + saline vs. U-0126 + saline; *t* = 6.08, *p* < 0.0001 for U-0126 + saline vs. U-0126 + KNT-127; Figure 5B) and in reexposure 2 (one-way ANOVA: *F*_(3,16)_ = 27.35, *p* < 0.0001; *post hoc* Bonferroni’s test: *t* = 7.018, *p* < 0.0001 for VEH + saline vs. VEH + KNT-127; *t* = 0.6176 for VEH + saline vs. U-0126 + saline; *t* = 5.727, *p* = 0.0002 for U-0126 + saline vs. U-0126 + KNT-127; Figure 5B). Figure 5C shows the time courses of freezing rates for each session. Two-way repeated-measures ANOVA (drug × session) revealed significant main effects of session (*F*_(2,32)_ = 37.0, *p* < 0.0001), drug (*F*_(3,16)_ = 22.53, *p* < 0.0001), and interaction between drug and time (*F*_(6,32)_ = 31.64, *p* < 0.0001; Figure 5C).

**FIGURE 6:**
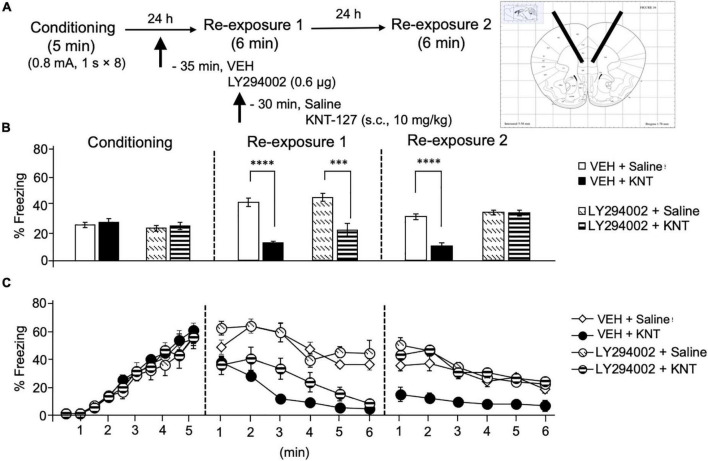
Intra-IL administration of the PI3K/Akt inhibitor abolished the fear-reducing effect of KNT-127. **(A)** Experimental design. Mice were subcutaneously administered with saline or KNT-127 (10 mg/kg) 30 min before reexposure 1 (6 min). Five minutes before injecting these drugs, a vehicle or a PI3K inhibitor, LY294002, was administered bilaterally into the IL (35 min before reexposure 1). Twenty-four hours after reexposure 1, a fear memory was tested in the same context for 6 min (reexposure 2). **(B)** Freezing rates of mice during conditioning, reexposures 1 and 2. LY294002 abolished the effect of KNT-127 during reexposure 2. The number of mice in each group was as follows: *n* = 6 for vehicle and subsequent saline (VEH + saline), *n* = 5 for vehicle and subsequent KNT-127 (VEH + KNT), *n* = 5 for LY294002 and subsequent saline (LY294002 + saline), and *n* = 6 for LY294002 and subsequent KNT-127 (LY294002 + KNT). **(C)** Time course of the freezing rates of mice during conditioning, reexposures 1 and 2. Data are expressed as the means ± SEM. Data were expressed as mean ± SEM. ***p < 0.001, ****p < 0.0001 for comparisons between KNT-127 and LY294002 treatment groups, by two-way ANOVA and *post hoc* Bonferroni’s test.

Next, we sought to clarify a molecular basis underlying the effect of KNT-127 in the IL. For this purpose, we focused on PI3K/Akt pathway, because it has been reported that PI3K/Akt pathway in the mPFC is activated along with the enhancement of fear extinction induced by ketamine administration (Girgenti et al., 2017). We administered VEH or a PI3K inhibitor, LY294002 (0.6 μg/mouse) to the IL 30 min before the administration of saline or KNT-127 (10 mg/kg, s.c., *n* = 6 for VEH + saline, *n* = 5 for VEH + KNT-127, *n* = 5 for LY294002 + saline, and *n* = 6 for LY294002 + KNT-127; Figure 6A). Figure 6B shows the total freezing rates of mice in each session. LY294002 in the IL produced no significant effects of KNT-127 in reexposure 1 (one-way ANOVA: *F*_(3,18)_ = 19.91, *p* < 0.0001; *post hoc* Bonferroni’s test: *t* = 5.96, *p* < 0.0001 for VEH + saline vs. VEH + KNT-127; *t* = 0.8167 for VEH + saline vs. LY294002 + saline; *t* = 4.792, *p* = 0.0009 for LY294002 + saline vs. LY294002 + KNT-127; Figure 6B), but LY294002 abolished the effect of KNT-127 in reexposure 2 (one-way ANOVA: *F*_(3,18)_ = 27.44, *p* < 0.0001; *post hoc* Bonferroni’s test: *t* = 6.982, *p* < 0.0001 for VEH + saline vs. VEH + KNT-127; *t* = 0.9309 for VEH + saline vs. LY294002 + saline; *t* = 0.04433 for LY294002 + saline vs. LY294002 + KNT-127; Figure 6B). Figure 6C shows the time courses of freezing rates for each session. Two-way repeated-measures ANOVA (drug × session) revealed significant main effects of session (*F*_(2,36)_ = 13.83, *p* < 0.0001), drug (*F*_(3,18)_ = 17.72, *p* < 0.0001), and interaction between drug and time (*F*_(6,36)_ = 16.52, *p* < 0.0001; Figure 6C).

These results suggest that the action of KNT-127 in the IL is mediated by PI3K/Akt, but not MEK/ERK signaling pathways.

### Administration of KNT-127 in the Prelimbic Cortex and Hippocampus Produced No Significant Effects on Contextual Fear Memory

**FIGURE 7:**
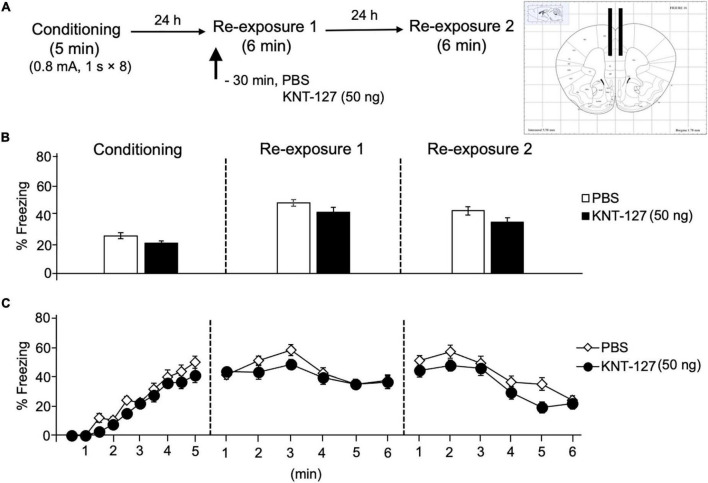
Intra-PL administration of KNT-127 produced no significant effects on contextual fear memory. **(A)** Experimental design. Mice were bilaterally administered with PBS (*n* = 12) or KNT-127 (*n* = 9 for 50 ng) into the PL 30 min before reexposure 1 (6 min). Twenty-four hours after reexposure 1, a fear memory was tested in the same context for 6 min (reexposure 2). **(B)** Freezing rates of mice during conditioning, reexposures 1 and 2. KNT-127 does not reduce freezing rates during reexposures 1 and 2 when administered into the PL. **(C)** Time course of the freezing rates of mice during conditioning, reexposures 1 and 2. Data are expressed as the means ± SEM.

Next, we also examined the contribution of another key brain regions in the fear circuit, PL and HPC, to the fear-reducing effect of KNT-127. We microinjected mice with KNT-127 in the PL at dose of 50 ng/mouse 30 min before the reexposure session 1 [PBS (*n* = 12) or KNT-127 (*n* = 9 for 50 ng); Figure 7A]. Figure 7B shows the total freezing rates of mice in each session. Intra-PL administration of KNT-127 did not produce a significant effect on the freezing rates during reexposures 1 and 2 when compared to PBS (*t*-test: *t*_(21)_ = 1.651, *p* = 0.1153 for reexposure 1; *t*_(21)_ = 1.76, *p* = 0.0945 for reexposure 2; Figure 7B). Figure 7C shows the time courses of freezing rates for each session. Two-way repeated-measures ANOVA (drug × session) revealed significant main effects of each session (*F*_(2,38)_ = 91.03, *p* < 0.0001), but not drug (*F*_(1,19)_ = 3.248, *p* = 0.0874) and interaction between drug and time (*F*_(2,38)_ = 1.069, *p* = 0.3535; Figure 7C).

**FIGURE 8:**
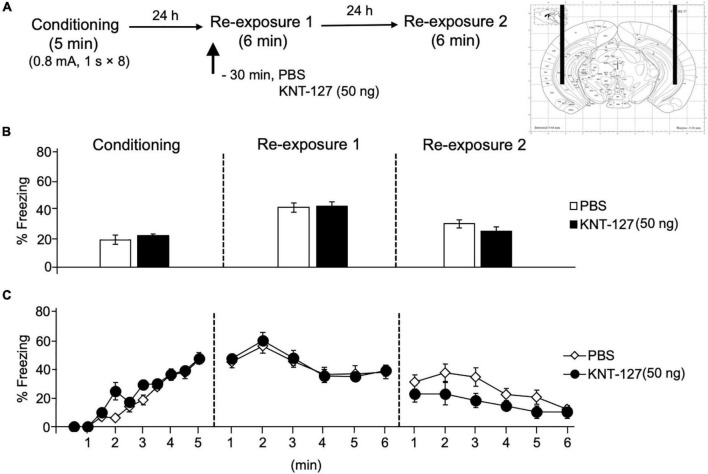
Intra-HPC administration of KNT-127 produced no significant effects on contextual fear memory. Intra-HPC administration of KNT-127 does not affect the extinction of contextual fear. **(A)** Experimental design. Mice were bilaterally administered with PBS (*n* = 14) or KNT-127 (*n* = 8 for 50 ng) into the HPC 30 min before reexposure 1 (6 min). Twenty-four hours after reexposure 1, a fear memory was tested in the same context for 6 min (reexposure 2). **(B)** Freezing rates of mice during conditioning, reexposures 1 and 2. KNT-127 does not reduce freezing rates during reexposures 1 and 2 when administered into the HPC. **(C)** Time course of the freezing rates of mice during conditioning, reexposures 1 and 2. Data are expressed as the means ± SEM.

We treated mice with microinfusions of KNT-127 in the HPC 30 min before the reexposure session 1 [PBS (*n* = 14) or KNT-127 (*n* = 8 for 50 ng)], (Figure 8A). Figure 8B shows the total freezing rates of mice in each session. Intra-HPC administration of KNT-127 did not produce a significant effect on the freezing rates during reexposures 1 and 2 when compared to PBS (*t*-test: *t*_(22)_ = 0.2258, *p* = 0.3357 for reexposure 1; *t*_(22)_ = 1.195, *p* = 0.6014 for reexposure 2; Figure 8B). Figure 8C shows the time courses of freezing rates for each session. Two-way repeated-measures ANOVA (drug × session) revealed significant main effects of each session (*F*_(2,40)_ = 34.89, *p* < 0.0001), but not drug (*F*_(1,20)_ = 0.01772) and interaction between drug and time (*F*_(2,40)_ = 1.406, *p* = 0.2570; Figure 8C).

These results suggest that intra-PL and HPC administrations of KNT-127 produced no significant effects on contextual fear memory.

## Discussion

This study shows that local administration of KNT-127 into the BLA and IL significantly reduced contextual fear memory in a DOP-dependent manner (Figures 1, 2, 4). In addition, the fear-reducing effect of systemic KNT-127 was mediated by MEK/ERK signaling pathways in the BLA (Figure 3), but not in the IL (Figure 5). Furthermore, PI3K/Akt signaling pathways in the IL mediate the effect of systemic KNT-127 (Figure 6). At the same time, no such effect was observed when KNT-127 was administrated into the PL and HPC (Figures 7, 8).

The results in this study suggest that KNT-127 in the BLA produced anxiolytic-like and extinction-facilitating effects during reexposure 1 and reexposure 2, respectively (Figures 1, 2). This notion is supported by our previous observation that SNC80, another anxiolytic DOP agonist, produced a significant reduction in the freezing behavior of mice only in reexposure 1, without effects on the freezing behaviors in reexposure 2 (Yamada et al., 2019), and also a benzodiazepine anxiolytic drug, diazepam (Sugiyama et al., 2015). In another behavioral analysis, KNT-127 and SNC80 exhibited robust anxiolytic-like effect in the elevated plus maze test (Saitoh et al., 2004, 2013). Based on these results, we proposed that a decrease in the freezing rate immediately after drug administration during reexposure 1 results from anxiolytic-like effects, whereas a decrease in the freezing rate in a drug-free state in reexposure 2 represents facilitation of fear extinction (Yamada et al., 2019). Another possibility is that KNT-127 inhibited fear memory retrieval in reexposure 1, because the freezing rates of mice at the beginning of reexposure 1 were significantly lower in the KNT-127 group than that in the control group. In addition, it is possible that KNT-127 facilitates retrieval of extinction memory of contextual fear in reexposure 2, not just facilitates extinction learning in reexposure 1.

In addition, we were not able to detect any effect of NTI application on extinction learning and retrieval at any stage, which suggest that synthetic activation of DOP is required to induce facilitation of fear extinction, and endogenous δ-opioid system may not contribute enough to the effect. However, in the elevated plus maze, NTI showed the decreases in the time spent in the open arm, which suggests the anxiogenic effect of NTI (Saitoh et al., 2004). These results arise the possibility that DOPs act differentially on anxiety-like behavior that can be detected in the elevated plus maze and fear conditioning test, respectively. Clarifying these points should be addressed in the future study.

In our experiments, control group seemed not fully extinguish the fear even at the end of extinction. It may be due to the length of reexposure (6 min). We aimed to show greater reduction in freezing response in the KNT-127-treated mice, and we used this short reexposure protocol to examine the fear extinction. Although this 6-min reexposure induced extinction of contextual fear in mice (Yamada et al., 2009), another extinction protocol with longer reexposure that achieves complete extinction in the control group would be helpful to examine the effect of KNT-127 on fear extinction in detail.

We previously reported that subcutaneous administration of KNT-127 increased p-ERK in the BLA 60 min after the extinction training (Yamada et al., 2019). Here, our results indicate that the extinction-facilitating effects of KNT-127 in the BLA were mediated by the MEK/ERK pathway (Figure 3). These results are consistent with the previous reports that KNT-127 increased p-ERK in the BLA. The MEK/ERK pathway inhibition in the BLA suppressed extinction both in fear conditioning and passive avoidance tests in mice (Herry et al., 2006; Fukushima et al., 2021). A recent study showed that another DOP agonist, SNC80, activates ERK1/2 in the BLA and reduces anxiety-like behavior in the elevated plus maze test and fear-potentiated startle response (Ko et al., 2021). These observations implicated that ERK signaling in the BLA may participate both in anxiety-like behavior and in fear extinction. Future studies should be conducted to examine the involvement of ERK-dependent signaling using genetic approaches, such as ERK siRNA or ERK-deficient mice. We propose that at least the BLA-mediated signaling pathway is essential for both the extinction-facilitating and anxiolytic-like effects of KNT-127.

Interestingly, when KNT-127 was microinjected into the IL, the freezing rate was significantly decreased only at reexposure 2 (Figure 4). This observation indicated that DOPs in the IL play a role only in the extinction phase, which is consistent with previous reports demonstrating that the inactivation of the IL with GABA_*A*_ receptor agonist muscimol inhibited fear extinction but not fear expression (Laurent and Westbrook, 2009; Sierra-Mercado et al., 2011) and that the activation of the IL enhanced fear extinction (Vargas et al., 2021). However, the observation that KNT-127 administered into the IL facilitates extinction in this study is inconsistent with our previous observation that no significant change in the level of p-ERK in the mPFC was observed after subcutaneous administration of KNT-127 (Yamada et al., 2019). Here, our results indicated that the extinction-facilitating effects of KNT-127 were mediated by PI3K/Akt pathway, but not MEK/ERK pathway in the IL (Figures 5, 6). Several reports have shown the involvement of such signaling pathways. For example, PI3K cascade in the IL is required to consolidate fear extinction memory (Kritman and Maroun, 2013). Also, ketamine has been reported to increase the protein kinase B (Akt) level that converges to regulate mTORC1 signaling in the mPFC during extinction sessions (Girgenti et al., 2017). Concerning the DOP, there are many connections between DOP and molecular mechanisms mediated by Akt (Olianas et al., 2011; Lv et al., 2017). *N*-desmethylclozapine (NDMC), a DOP agonist, exerts neuroprotective effects by activating Gi/Go-coupled DOP and stimulating Akt signaling in Chinese hamsters ovary cells transfected with the human δ-opioid receptor (CHO/DOR) (Olianas et al., 2011). These results are consistent with our result in this study. However, we have no direct connection between this signaling pathway and DOP, and further experiments should be conducted to clarify the molecular targets that mediate the effect of KNT-127 downstream of DOP in each brain region.

Unlike IL, KNT-127 administration into the PL did not affect the freezing rate of mice at all (Figure 7). The results suggest that DOPs in the PL affect neither anxiety-like behavior nor extinction learning observed in the contextual fear conditioning test. On the other hand, we previously reported that intra-PL perfusion of KNT-127 suppresses anxiety-like behavior in the open-field test (Saitoh et al., 2018). Our previous findings are inconsistent with the present results that KNT-127 did not show the anxiolytic-like effects in reexposure 1. Therefore, we speculate that the neural circuits of DOPs in the PL differ between the innate anxiety of mice caused by novel open-field apparatus and the anxiety of mice caused by the acquisition of fear memory.

Despite our previous result that the p-ERK level in the HPC was upregulated after the extinction training (Yamada et al., 2019), KNT-127 administered into the HPC produced no significant effects on extinction of contextual fear memory (Figure 8). Therefore, it is possible that the increase of p-ERK in the HPC by KNT-127 is not involved in the expression of the extinction-facilitating effect in the behavioral tests. Recently, it has been reported that the increase of p-CREB after upregulation of p-ERK is observed in the HPC when fear extinction successfully occurred (Mamiya et al., 2009; Fukushima et al., 2021). Therefore, it is possible that the upregulation of p-ERK in the HPC after subcutaneous administration of KNT-127 is insufficient to affect fear extinction. To determine the molecular mechanism underlying KNT-127-induced enhancement of extinction, an assessment of the level of p-CREB may be more suitable to be addressed.

**FIGURE 9:**
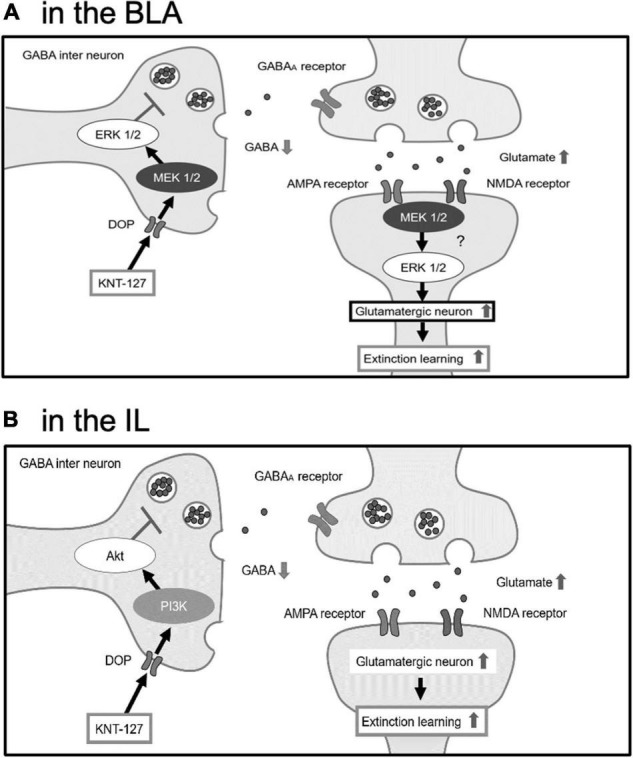
The hypothesis that BLA and IL facilitate fear extinction through different signaling pathways. **(A)** In the BLA, KNT-127 exerts extinction-facilitating effect *via* ERK/MEK pathway. **(B)** In the IL, KNT-127 exerts extinction-facilitating effect *via* PI3K/Akt pathway. KNT-127 acting on DOPs on GABAergic interneuron in the BLA and IL produce the disinhibition of glutamatergic neurons.

Electrophysiological experiments revealed that endogenous and synthetic agonists of DOP, such as methionine-enkephalin (Met-Enk), [D-Pen^2^,D-Pen^5^]Enkephalin (DPDPE), and SNC80 bidirectionally act on excitatory or inhibitory synaptic transmissions in the central synapses. For example, Met-Enk and SNC80 significantly decreased excitatory postsynaptic current (EPSC) in the nucleus of solitary tract (Shen and Johnson, 2002; Zhu et al., 2009). DPDPE inhibited EPSC in the spinal lamina II (Glaum et al., 1994). On the other hand, DPDPE inhibits GABA release in the HPC and decreased the frequency of miniature inhibitory postsynaptic current (mIPSC) in the subthalamic nuclei and also Met-Enk (Lupica, 1995; Mańko et al., 2011). Furthermore, DPDPE disinhibited the EPSC by suppressing local feed-forward GABA signaling in the anterior cingulate cortex (Birdsong et al., 2019). For KNT-127, we previously demonstrated the similar results outside of the amygdala. Bath-application of KNT-127 significantly reduced the frequency of EPSC and decreased the number of action potentials induced by current injection in the mPFC (Yamada et al., 2021). Although we have no such electrophysiological evidence in the amygdala and IL, it is possible that KNT-127 produces the decrease in the transmitter release and neuronal excitability in these brain regions. Taking our results and previous pieces of evidence into account, we hypothesize a plausible mechanism of action for KNT-127 on fear extinction (Figure 9). Our speculation is as follows: KNT-127 binding to DOPs on GABAergic neurons in the BLA and IL may inhibit GABA release and results in disinhibition of glutamatergic neurons to stimulate glutamate release from the presynaptic site. Although MEK/ERK signaling in the BLA and PI3K/Akt signaling in the IL may be activated during this activation of glutamatergic neurons. It is possible because there is a report demonstrated the colocalization of DOPs and GABA in the brain (Tongjaroenbuangam et al., 2006). In the BLA, as previously reported, there are glutamatergic neurons called “extinction neurons,” specifically activated by extinction learning (Herry et al., 2008). Therefore, DOPs located on the GABAergic interneurons, which govern these extinction neurons in the BLA, are the candidates of the target of KNT-127. Regarding the involvement of the IL, the neurotransmission from the IL to GABAergic intercalated cells (ITC) located between the BLA and central nucleus of the amygdala (CeA) is essential for fear extinction (Likhtik et al., 2008; Amano et al., 2010; Amir et al., 2011). Furthermore, endogenous ligand of DOP, Met-enkephalin, reduces the strength of BLA synaptic inputs to the intercalated nucleus of the amygdala (Im, more ventral portion of the ITC), and it could allow Im neurons to be more strongly influenced by the input from the cortex, such as the IL (Winters et al., 2017). If KNT-127 enhances this circuit, both BLA-lateral CeA (CeL) and IL-ITC (Im) neural circuits are activated to inhibit the medial CeA (CeM), the output center of the amygdala, which results in suppressing the freezing behavior (Mańko et al., 2011).

One of the other types of the opioid receptor, μ-opioid receptor, predominantly distribute in the ITC, but much less in the BLA (Likhtik et al., 2008). This differential expression pattern, functional similarity with DOP was demonstrated. GABAergic, but not glutamatergic transmission between medial ITC and CeM, is attenuated by a μ-opioid receptor agonist, DAMGO (Blaesse et al., 2015). Endogenously released opioids inhibit glutamate release through the DOPs presynaptically. Postsynaptically, the opioids activate a potassium conductance through the μ-opioid receptor, which suggests that endogenously released opioids directly regulate neuronal excitability (Winters et al., 2017). Therefore, the way of synaptic modulation by μ-opioid system in the amygdala may be helpful to understand underlying mechanism of extinction-facilitating action of KNT-127. Future study should address the hypothesis of the mechanism of action of KNT-127 in the neural circuit of fear memory regulation.

## Conclusion

We found that a selective agonist of DOP, KNT-127, facilitated the extinction of contextual fear memory *via* DOPs and MEK/ERK signaling in the BLA and PI3K/Akt signaling in the IL. We propose that the effect of KNT-127 is mediated by distinct signaling pathways in different brain regions.

## Data Availability Statement

The datasets supporting the conclusions of this article are available on request to the corresponding author.

## Ethics Statement

The animal study was reviewed and approved by the Institutional Animal Care and Use Committee of the Tokyo University of Science (approval nos. Y19032, Y20020, and Y21002).

## Author Contributions

DY and AS designed and supervised the project. AK, DY, SY, and MS conducted the experiments and analyzed the data. DY, AS, and AK wrote the manuscript. KI and HN synthesized and provided resources used in the study. All authors discussed the results and commented on the manuscript.

## Conflict of Interest

The authors declare that the research was conducted in the absence of any commercial or financial relationships that could be construed as a potential conflict of interest.

## Publisher’s Note

All claims expressed in this article are solely those of the authors and do not necessarily represent those of their affiliated organizations, or those of the publisher, the editors and the reviewers. Any product that may be evaluated in this article, or claim that may be made by its manufacturer, is not guaranteed or endorsed by the publisher.
